# Data on effects, tolerability and safety of Omega-3 Fatty Acids in Enteral Nutrition in the Critically ill

**DOI:** 10.1016/j.dib.2018.10.017

**Published:** 2018-10-12

**Authors:** W.A.C. Koekkoek, V. Panteleon, A.R.H. van Zanten

**Affiliations:** a Department of Intensive Care Medicine, Gelderse Vallei Hospital, Willy Brandtlaan 10, 6716 RP Ede, the Netherlands; b Wageningen University, 6708 PB Wageningen, the Netherlands; c Department of Intensive Care Medicine, Gelderse Vallei Hospital, Willy Brandtlaan 10, 6716 RP Ede, the Netherlands

## Abstract

In addition to the data reported in our systematic review and meta-analysis ‘Current Evidence on Omega-3 Fatty Acids in Enteral Nutrition in the Critically ill’ we present data on intensive care unit and hospital mortality, age distribution between included studies, tolerability and adverse events of enteral omega-3 supplementation compared with control interventions in the critically ill. Moreover, we report additional analyses on 28-day mortality comparing old versus new studies and high versus low quality trials. Finally, we report baseline and follow-up levels of eicosapentaenoic acid (EPA) and docosahexaenoic acid (DHA) reported in the trials included in Koekkoek et al. (2018). For further interpretation and discussion we recommend reading our systematic review and meta-analysis Current Evidence on Omega-3 Fatty Acids in Enteral Nutrition in the Critically ill’.

**Specifications table**

**Table:** 

Subject area	Medicine
More specific subject area	Analysis of data on omega-3 fatty acids in critical care nutrition
Type of data	Tables and figures
How data was acquired	Data were acquired through meta-analysis of 24 RCT׳s on enteral omega-3 fatty acid supplementation in critically ill patients
Data format	Analyzed
Experimental factors	Critically ill patients receiving enteral omega-3 fatty acid supplementation included in randomized controlled trials were included
Experimental features	Forest plots were generated and relative risk ratio׳s calculated to show the effects of omega-3 fatty acid supplementation
Data source location	A systematic review was conducted to identify all relevant randomized clinical trials published before January 2018 in MEDLINE, Embase, CINAHL and the Cochrane Central Register of Controlled Trials.
Data accessibility	A systematic review was conducted to identify all relevant randomized clinical trials published before January 2018 in MEDLINE, Embase, CINAHL and the Cochrane Central Register of Controlled Trials.

**Value of the data**

• The data on tolerability and adverse events are helpful in a risk-benefit analysis of omega-3 supplementation in the critically ill.
• Data on ICU and hospital mortality provide more information on safety of omega-3 interventions.
• Data on age distribution between studies may be helpful in interpretation of results and may give rise to specific treatment of age-groups.
• Data on EPA and DHA baseline and follow-up levels may be of value in interpretation of the effects of omega-3 supplementation as they may be related to serum levels.

## 1 Data

**Fig. 1:**
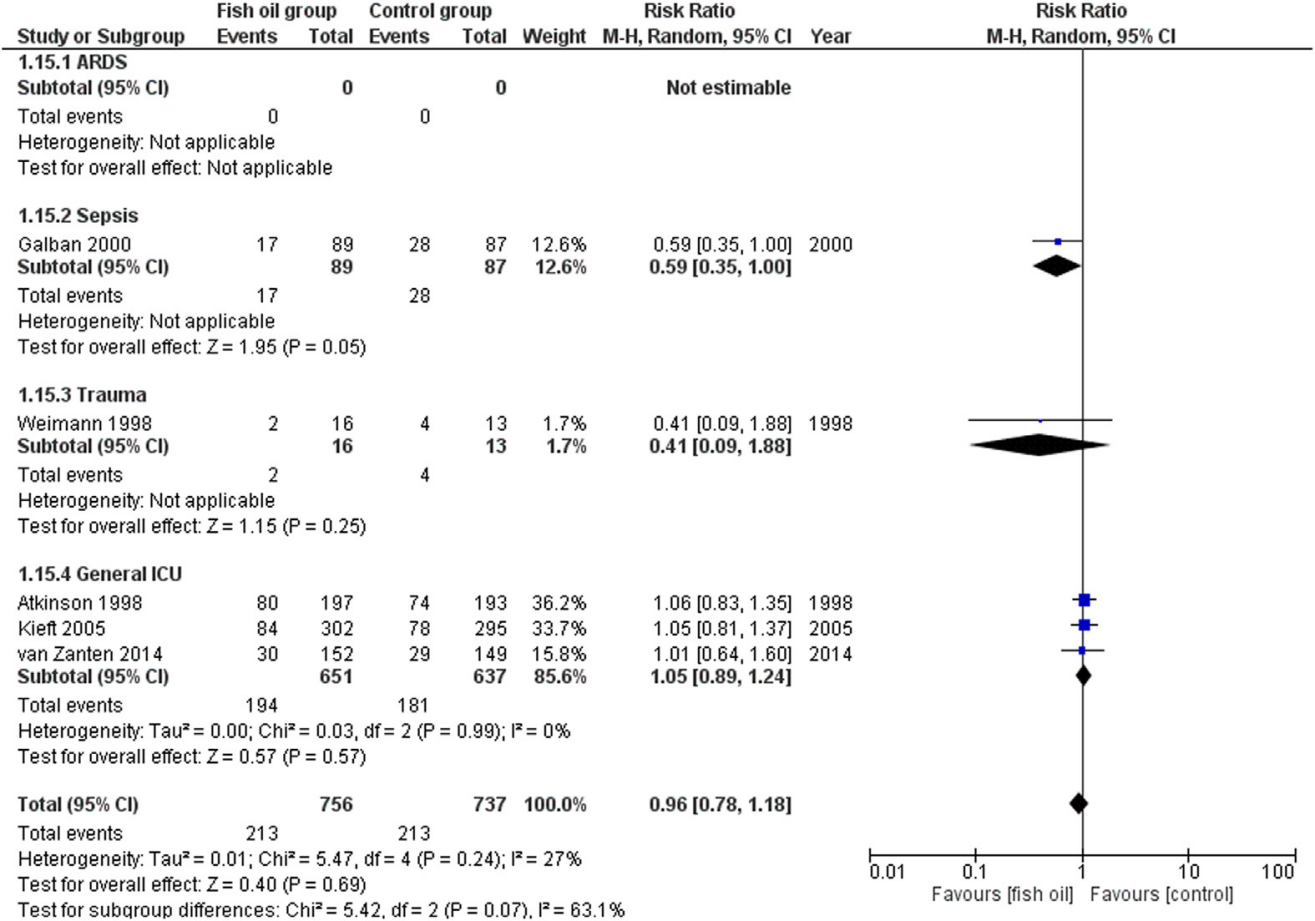
The effects of fish oil supplementation on ICU mortality in different ICU populations. ARDS: Acute respiratory distress syndrome; ICU: Intensive Care Unit; CI: confidence interval.

**Fig. 2:**
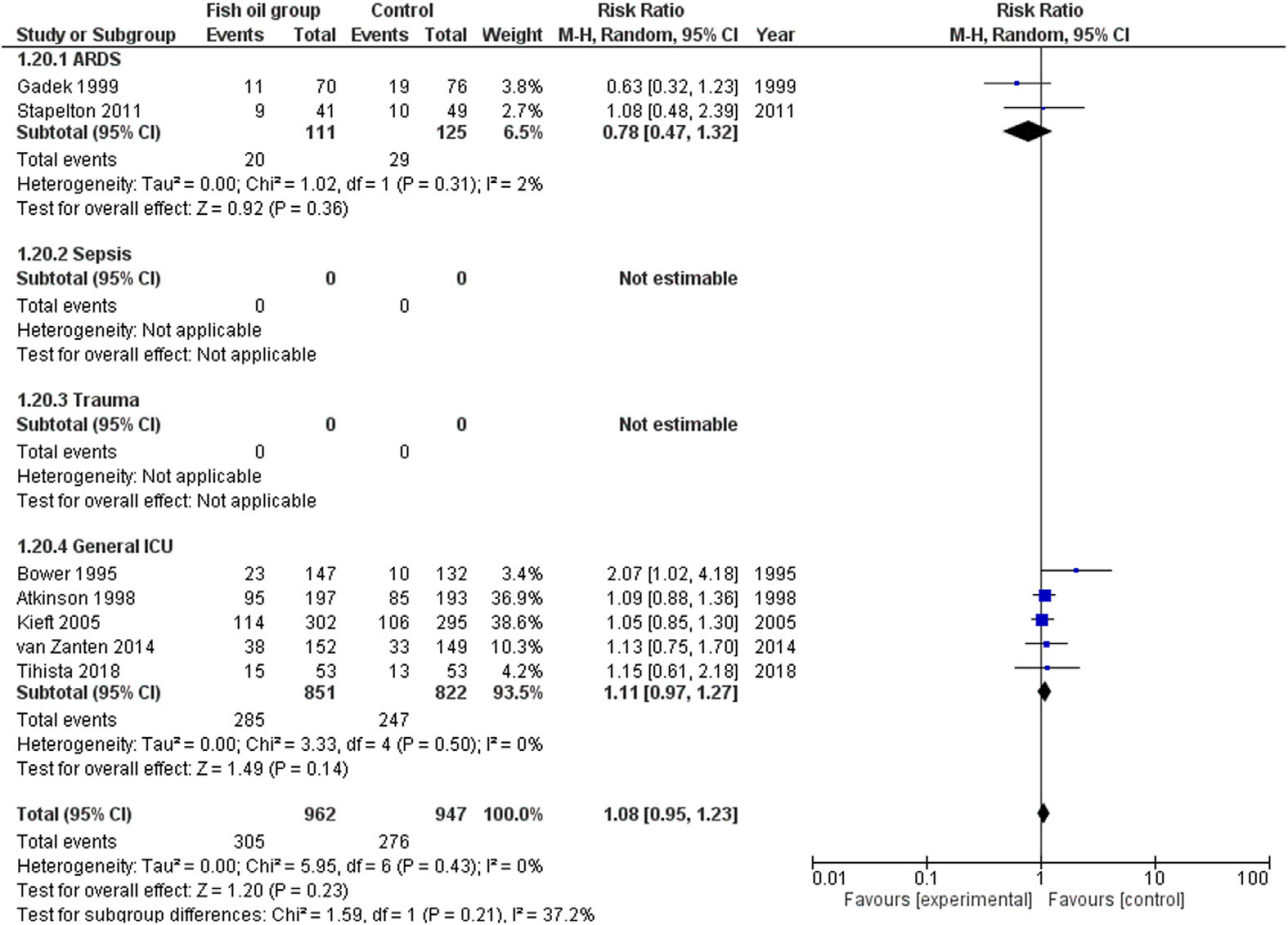
The effects of fish oil supplementation on hospital mortality in different ICU populations. ARDS: Acute respiratory distress syndrome; ICU: Intensive Care Unit; CI: confidence interval.

**Fig. 3:**
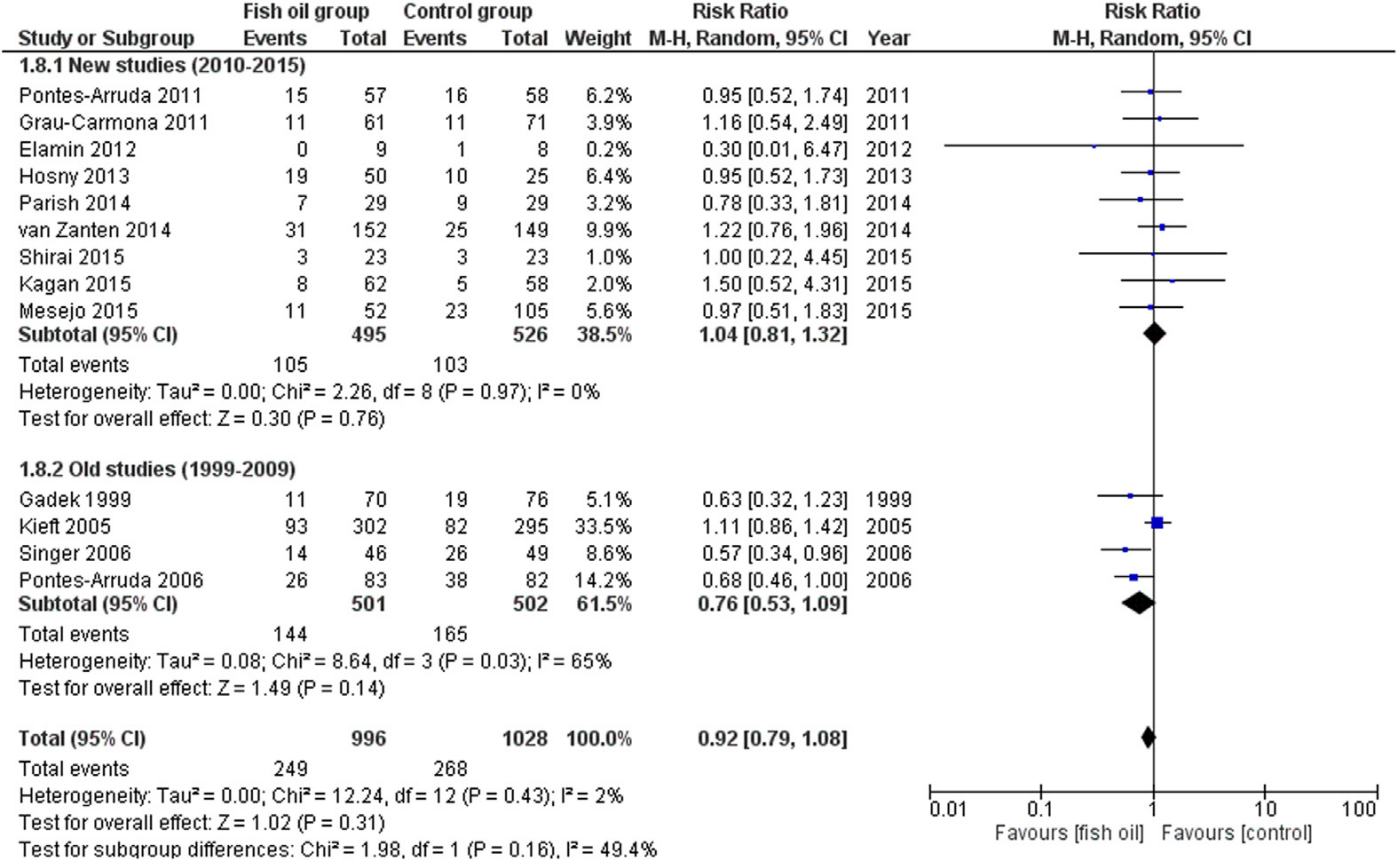
The effects of fish oil supplementation on 28-day mortality, comparing old versus new RCTs. CI: confidence interval.

**Fig. 4:**
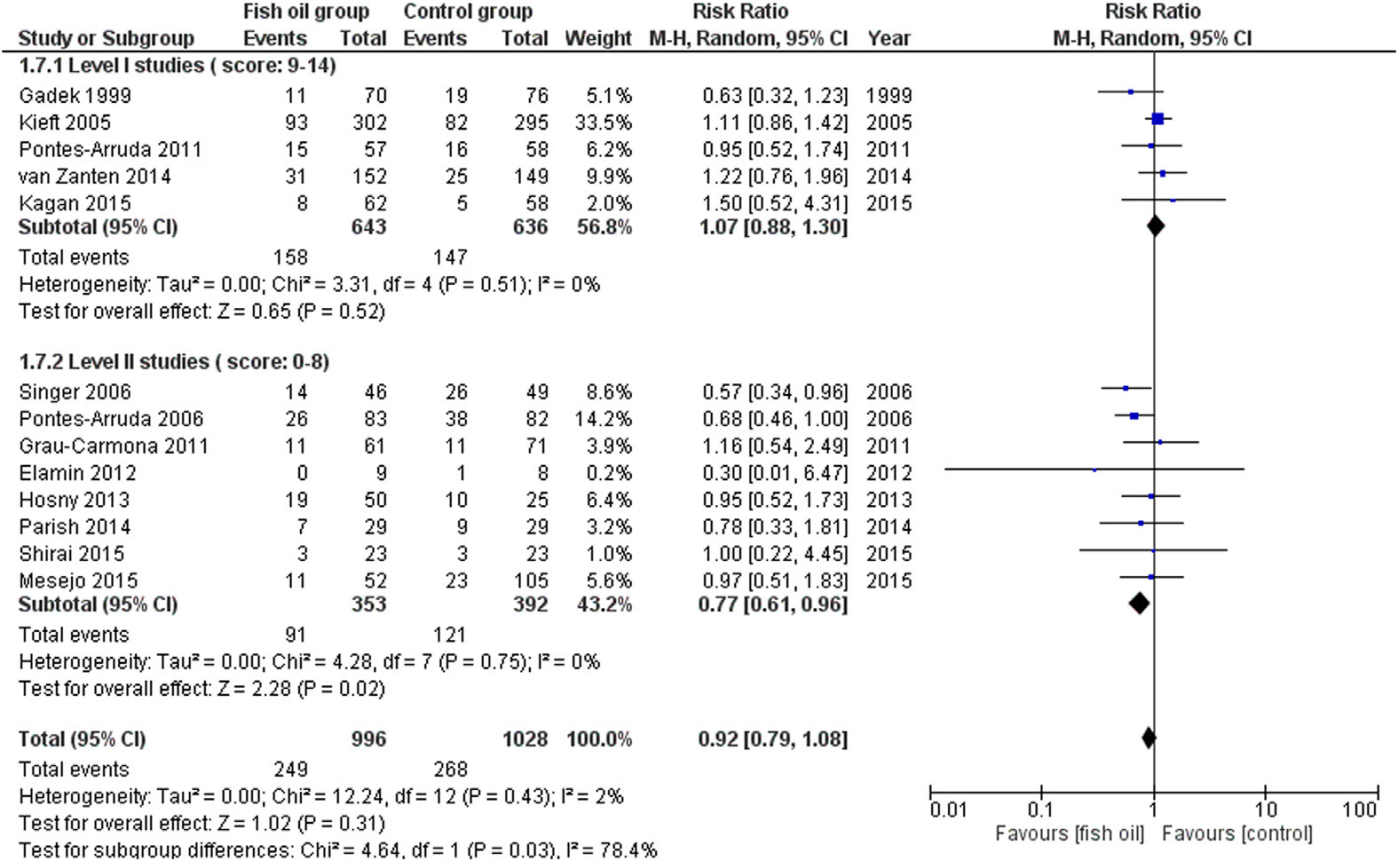
The effects of fish oil supplementation on 28-day mortality, comparing high versus low quality RCTs. CI: confidence interval.

We share data regarding ICU and hospital mortality, age distribution between included studies in [1], tolerability and adverse events of enteral omega-3 supplementation compared with placebo in the critically ill. Fig. 1 shows the effects of fish oil supplementation on ICU mortality in different ICU populations. Fig. 2 shows the effects of fish oil supplementation on hospital mortality in different ICU populations. In Fig. 3 the effects of fish oil supplementation on 28-day mortality, comparing old versus new randomized controlled trials (RCTs) are reported. In Fig. 4 the effects of fish oil supplementation on 28-day mortality, comparing high versus low quality RCTs are shown.

**Fig. 5:**
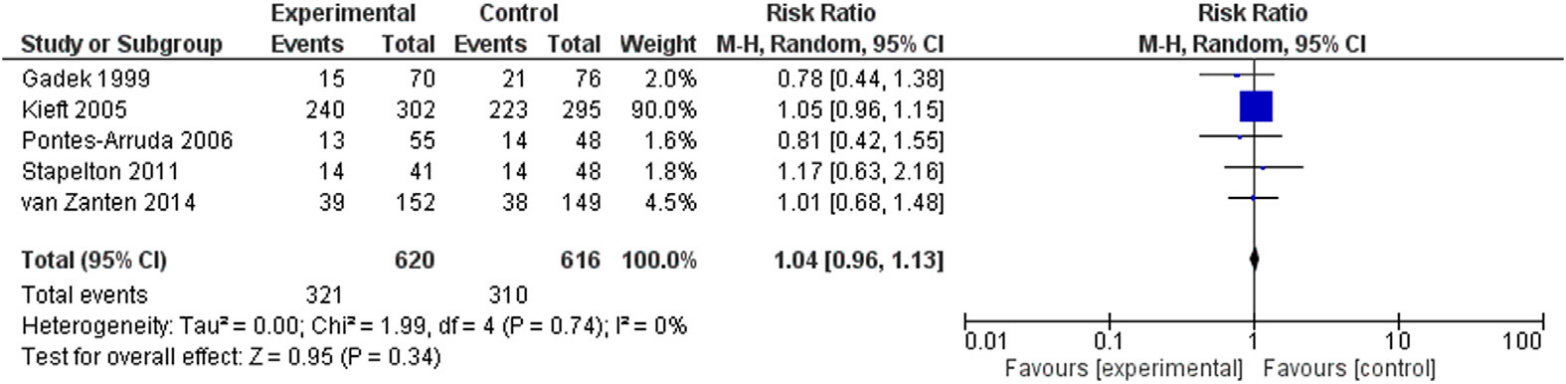
Adverse events in RCTs comparing fish oil supplementation with control enteral feeding. CI: confidence interval.

Adverse events registered in the trials included in [1] are reported in Fig. 5.

Table 1 reports baseline and follow-up EPA and DHA levels. Furthermore, Table 2 reports on age distribution in the studies included in [1]. Additionally, tolerability of omega-3 supplementation in ICU patients is reported in Table 3.

**Table 1:** EPA and DHA levels before and after fish oil supplementation.

Study	Baseline EPA levels	Baseline DHA levels	Reached EPA levels	Reached DHA levels	Remarks
Atkinson 1998	NR	NR	NR	NR	
Bower 1995	NR	NR	Intervention group: 97% increase, *p* < 0.01	Intervention group: 72% increase, *p* < 0.01	Subset of 72 random patients
			Control group: NS difference	Control group: NS difference	
Elamin 2012	NR	NR	NR	NR	
Gadek 1999	Intervention group: 0.4% of plasma phospholipid fatty acids	NR	Intervention group day 4:6.9% of plasma phospholipid fatty acids, *p* < 0.001	NR	Extracted from figures
	Control group: 0.6% of plasma phospholipid fatty acids				
			Intervention group day 7:8.4% of plasma phospholipid fatty acids, *p* < 0.001		
			Control group day 4 and 7:0.1% of plasma phospholipid fatty acids, NS		
Galban 2000	NR	NR	NR	NR	
Grau-Carmo NR 2011	NR	NR	NR	NR	
Hosny 2013	NR	NR	NR	NR	
Jakob 2017	NR	NR	NR	NR	
Kagan 2015	Intervention group: 5.5 ± 1.5%		Intervention group day 4: 6.0 ± 1.2%		Omega-3 Index
	Control group: 4.6 ± 0.9%		Intervention group day 8: 6.8 ± 1.0%		Analyzed only for patients completing 8 days of the study (*n* = 33 in the control group and *n* = 40 in study group)
			Control group day 4: 4.4 ± 0.8%		
			Control group day 8: 4.5 ± 0.8 %		
Kieft 2005	NR	NR	NR	NR	
Kudsk 1996	NR	NR	NR	NR	
Mendez 1997	NR	NR	NR	NR	
Mesejo 2015	NR	NR	NR	NR	
Parish 2014	NR	NR	NR	NR	
Pontes-Arruda 2006	NR	NR	NR	NR	
Pontes-Arruda 2011	NR	NR	NR	NR	
Rice 2011	Intervention group: 2 mg/L	NR	Intervention group day 3, 6 and 12: approximately 16 mg/L	NR	Extracted from text and figure. Levels were measured in the first 60 patients. Because of unavailable samples, actual measurements are from 24 *n*−3 and 30 control patients at baseline (24 in each group at day 3, 17 in each group on day 6, and 8 *n*−3 and 9 control patients on day 12).
	Control group: 2 mg/L				
			Control group day 3, 6 and 12: approximately 2 mg/L		
Shirai 2015	NR	NR	NR	NR	
Singer 2006	NR	NR	NR	NR	
Stapelton 2011	NR	NR	Intervention group day 5: 31.9 mg/L (IQR 24.1–59.7)	Intervention group day 5: 24.1 mg/L (IQR 15.8–39.8)	
			Control group day 5:2.4 mg/L (IQR 1.5–6.3)	Control group day 5: 12.8 mg/L (IQR 9.1–17.9)	
Thiella 2012	NR	NR	NR	NR	
Tihista 2017	NR	NR	NR	NR	
Weimann 1998	NR	NR	NR	NR	
Van Zanten 2014	Intervention group: (EPA+DHA):LCP ratio = 0.03 ± 0.01		Intervention group day 4: (EPA+DHA):LCP ratio increase of 3.4%(95% CI, 3.0–3.8%)		LCP = long-chain polyunsaturated fatty acids
	Control group: (EPA+DHA):LCP ratio = 0.03 ± 0.01		Intervention group day 8: (EPA+DHA):LCP ratio increase of 5.1%(95% CI, 4.6–5.5%)		
			Control group day 4: (EPA+DHA):LCP ratio increase of −0.3%(95% CI, −0.4% to 0.2%)		
			Control group day 8: (EPA+DHA):LCP ratio increase of −0.5% (95% CI, −0.6% to 0.4%)		

EPA: eicosapentaenoic acid; DHA: docosahexaenoic acid; NR: not reported; NS: non-significant; IQR: interquartile range; LCP: long-chain polyunsaturated fatty acids.

**Table 2:** Age of study population of included RCTs.

Study	Age intervention	Age control
Atkinson 1998	63 (18–99)*	62 (18–87)*
Bower 1995	39 ± 18.2	39.9 ± 18.2
Elamin 2012	50.0 ± 22.2	55.2 ± 16.5
Gadek 1999	51 ± 2	51 ± 3
Galban 2000	53.9 ± 18.5	57.7 ± 16.9
Grau-Carmona 2011	62 (40–71)^	65 (51–76)^
Hosny 2013	52.8 ± 18.87 (intervention group A)	50.5 ± 14.77
	53.1 ± 12.47 (intervention group B)	
Jakob 2017	65.3 (52.6–75.3)^	61.6 (48.6–71.3)^
Kagan 2015	42.9 ± 18.6	38.4 ± 16.8
Kieft 2005	66.0 (49–74)^	68.0 (55–74)^
Kudsk 1996	34.3 ± 3.1^#^	31.8 ± 2.3^#^
Mendez 1997	28.2 ± 1.2^#^	35.3 ± 2.3^#^
Mesejo 2015	57 (43–70)^	60 (45–71)^
		58 (46–68)^
Parish 2014	64.4 ± 10.2	62.7 ± 13.7
Pontes-Arruda 2006	64.3 ± 18.7	66 ± 20
Pontes-Arruda 2011	70 (64–78)^	72 (65–82)^
Rice 2011	55.5 ± 17.0	52.9 ± 16.5
Shirai 2015	71 (66–77)^	74 (60–80)^
Singer 2006	57.0 ± 18.7	62.3 ± 17.2
Stapelton 2011	49.0 ± 16.5	50.7 ± 16.5
Thiella 2012	49.3 ± 20.7	53.1 ± 19.3
Tihista 2017	38.7 ± 16.2	41.6 ± 16.6
Weimann 1998	34.8 ± 16.9	31.0 ± 11.8
Van Zanten 2014	57 ± 19	59 ± 18

Age is reported in mean ± standard deviation unless otherwise specified. * median with range ^ median with interquartile range ^#^ mean ± standard error of the mean.

**Table 3:** Tolerability of enteral fish oil supplementation.

Study		Nausea/ vomiting	Dyspepsia	High GRV	Aspiration	Diarrhea	Constipation	Abdominal distention	Ileus	Pancreatitis	Calories delivered	Replace tube	Achieved feeding target	Triglycerides	Prokinetics		Overall GI complications
Atkinson 1998	*Intervention*	NR	NR	NSD	NR	NSD	NR	NR	NR	NR	14 (0–32)^a^ kcal/kg/day	NR	50/197	NR		NR	NR
	*Control*	NR	NR	NSD	NR	NSD	NR	NR	NR	NR	13 (0–32)^a^ kcal/kg/day	NR	51/193	NR		NR	NR
Bower 1995	*Intervention*	NSD	NR	NR	1/147	NSD	NR	NR	NSD	NR	NR	NSD	100/147	NR		NR	NR
	*Control*	NSD	NR	NR	1/132	NSD	NR	NR	NSD	NR	NR	NSD	100/132	NR		NR	NR
Elamin 2012	*Intervention*	NR	NR	NR	NR	NR	NR	NR	NR	NR	NR	NR	9/9	NR		NR	NR
	*Control*	NR	NR	NR	NR	NR	NR	NR	NR	NR	NR	NR	8/8	NR		NR	NR
Gadek 1999	*Intervention*	1/70	0/70	NR	1/70	5/70	NR	2/70	1/70	2/70	NR	1/70	50/70	28% decrease on day 7		NR	NR
	*Control*	1/76	1/76	NR	0/76	5/76	NR	4/76	1/76	1/76	NR	5/76	43/76	19% decrease on day 7		NR	NR
Galban 2000	*Intervention*	NR	NR	NR	NR	20/89	NR	NR	NR	NR	1231 ± 411 kcal/day	NR	NR	NR		NR	48/89
	*Control*	NR	NR	NR	NR	12/87	NR	NR	NR	NR	1414 ± 471 kcal/day	NR	NR	NR		NR	56/87
Grau-Carmona 2011	*Intervention*	NR	NR	220 episodes/1000 days of EN	NR	271 episodes/1000 days of EN	NR	NR	NR	NR	1718 (1189–1956) kcal/day	NR	NSD	120.3 ± 40.3 mg/dL		NR	NR
	*Control*	NR	NR	279 episodes/1000 days of EN	NR	302 episodes/1000 days of EN	NR	NR	NR	NR	1599 (1351–1976) kcal/day	NR	NSD	106.9 ± 41.9 mg/dL		NR	NR
Hosny 2013	*Intervention*	NR	NR	NR	NR	5/25	NR	NR	NR	NR	NR	NR	NR	NR		NR	NR
	*Control*	NR	NR	NR	NR	4/25	NR	NR	NR	NR	NR	NR	NR	NR		NR	NR
Jakob 2017	*Intervention*	NR	NR	NR	NR	29/45	NR	NR	NR	NR	18.0 (12.5–20.9) kcal/kg/day	NR	NR	NR		15/45	NR
	*Control*	NR	NR	NR	NR	31/44	NR	NR	NR	NR	19.7 (17.3–23.1) kcal/kg/day	NR	NR	NR		15/44	NR
Kagan 2015	*Intervention*	NR	NR	NR	NR	1.1 ± 2.3 episodes	NR	NR	NR	NR	1612.8 ± 532.6 kcal/day	NR	52/62	NR		NR	NR
	*Control*	NR	NR	NR	NR	1.6 ± 4.4 episodes	NR	NR	NR	NR	1622.9 ± 728 kcal/day	NR	52/58	NR		NR	NR
Kieft 2005	*Intervention*	NR	NR	NR	NR	NR	NR	NR	NR	NR	NR	NR	NR	NR		NR	NR
	*Control*	NR	NR	NR	NR	NR	NR	NR	NR	NR	NR	NR	NR	NR		NR	NR
Kudsk 1996	*Intervention*	NR	NR	NR	NR	NSD	NR	NSD	NR	NR	18.03 ± 1.62 kcal/kg/day	NR	NR	NSD on day 7		NR	13/16
	*Control*	NR	NR	NR	NR	NSD	NR	NSD	NR	NR	18.29 ± 1.60 kcal/kg/day	NR	NR	NSD on day 7		NR	16/17
Mendez 1997	*Intervention*	NR	NR	NR	NR	150-400 mL/day	NR	NR	NR	NR	29.5 ± 2.6 kcal/kg/day	NR	NR	NR		NR	NR
	*Control*	NR	NR	NR	NR	300-700 ml/day	NR	NR	NR	NR	26.5 ± 3.2 kcal/kg/day	NR	NR	NR		NR	NR
Mesejo 2015	*Intervention*	NR	NR	1/52	NR	2/52	NR	0/52	NR	NR	22.2 ± 4.1 kcal/kg/day	NR	NR	NSD		NR	NR
	*Control*	NR	NR	3/105	NR	4/105	NR	1/105	NR	NR	21.7 ± 4.8 kcal/kg/day	NR	NR	NSD		NR	NR
Parish 2014	*Intervention*	NR	NR	NSD	NR	NSD	NR	NR	NR	NR	NSD	NR	NR	NR		NR	NR
	*Control*	NR	NR	NSD	NR	NSD	NR	NR	NR	NR	NSD	NR	NR	NR		NR	NR
Pontes-Arruda 2006	*Intervention*	0/55	1/55	NR	NR	9/55	NR	NR	NR	0/55	1621 ± 48 kcal/day	2/55	14/55	NR		NR	NR
	*Control*	1/48	0/48	NR	NR	7/48	NR	NR	NR	1/48	1647 ± 74 kcal/day	4/48	23/48	NR		NR	NR
Pontes-Arruda 2011	*Intervention*	2/53	NR	NR	NR	4/53	NR	NR	NR	NR	1538 (1295–1890) kcal/day	NR	NR	NR		NR	NR
	*Control*	3/53	NR	NR	NR	7/53	NR	NR	NR	NR	1523 (1370–1950) kcal/day	NR	NR	NR		NR	NR
Rice 2011	*Intervention*	3.8%	NR	3.2%	NR	28.7%	NR	9.3%	NR	NR	NR	NR	NR	NR		NR	NR
	*Control*	2.4%	NR	4.0%	NR	20.9%	NR	7.4%	NR	NR	NR	NR	NR	NR		NR	NR
Shirai 2015	*Intervention*	0/23	NR	NR	NR	6/23	NR	0/23	NR	NR	18.78 (18.12–20.21) kcal/kg/day	NR	NR	110 (94–131) mg/dL on day 14		NR	NR
	*Control*	0/23	NR	NR	NR	4/23	NR	0/23	NR	NR	19.48 (15.73–20.68) kcal/kg/day	NR	NR	92 (79–123) mg/dL on day 14		NR	NR
Singer 2006	*Intervention*	NR	NR	NR	NR	NR	NR	NR	NR	NR	1624 ± 512 kcal/day	NR	46/46	NR		NR	NR
	*Control*	NR	NR	NR	NR	NR	NR	NR	NR	NR	1420 ± 437 kcal/day	NR	49/49	NR		NR	NR
Stapelton 2011	*Intervention*	NR	NR	NR	NR	NR	NR	NR	NR	NR	7362 ± 3800 kcal/week	NR	NR	NR		NR	NR
	*Control*	NR	NR	NR	NR	NR	NR	NR	NR	NR	7495 ± 3831 kcal/week	NR	NR	NR		NR	NR
Thiella 2012	*Intervention*	NR	NR	NR	NR	NR	NR	NR	NR	NR	NR	NR	NR	NR		NR	NR
	*Control*	NR	NR	NR	NR	NR	NR	NR	NR	NR	NR	NR	NR	NR		NR	NR
Tihista 2017	*Intervention*	NR	NR	4/47	NR	2/47	45/47	NR	NR	NR	16 ± 4 kcal/kg/day	NR	NR	NR		NR	NR
	*Control*	NR	NR	15/45	NR	7/45	40/45	NR	NR	NR	17 ± 3 kcal/kg/day	NR	NR	NR		NR	NR
Weimann 1998	*Intervention*	NR	NR	466 ± 262 mL	NR	4/16	3/16	NR	NR	NR	561 ± 266 kcal/day	1 (1–4)	NR	NR		NR	NR
	*Control*	NR	NR	513 ± 154 mL	NR	4/13	6/13	NR	NR	NR	520 ± 342 kcal/day	1 (1–3)	NR	NR		NR	NR
Van Zanten 2014	*Intervention*	NR	NR	NR	NR	1/152	NR	NR	NR	NR	NR	NR	NR	NR		NR	NR
	*Control*	NR	NR	NR	NR	0/149	NR	NR	NR	NR	NR	NR	NR	NR		NR	NR

GRV: gastric residual volume; GI: gastro-intestinal; NR: not reported; NSD: non-significant difference; IQR: interquartile range; EN: enteral nutrition.

a IQR with range; NR = not reported; NSD = no significant difference, no absolute numbers reported in article.

## 2 Experimental design, materials and methods

The search strategy, study identification and selection criteria used to acquire the data presented in this article are reported in [1].
