# Supplementary material for: Data on effects, tolerability and safety of Omega-3 Fatty Acids in Enteral Nutrition in the Critically ill
Source: Data Brief. 2018 Oct 12;21:604–15. doi: 10.1016/j.dib.2018.10.017 (PMC6205069; doi:10.1016/j.dib.2018.10.017)
Supplement: Supplementary file 1 — Supplementary material [file mmc1.docx]

Conflict of Interest Statement and Funding sources

Arthur van Zanten reported that he has received honoraria for advisory board meetings, lectures, and travel expenses from\ Abbott, Baxter, BBraun, Beacon, Cardinal Health, Danone-Nutricia, Fresenius Kabi, Lyric and Nestle -Novartis. Inclusion fees for patients in nutrition trials were paid to the local ICU research foundation. The remaining authors have disclosed that they do not have any conflicts of interest.

There was no funding source for this work.
